# Utilization of Fruit Juice Processing Wastes as Prebiotic Ingredients in Probiotic Yogurt: Effects on Microbial Short Chain Fatty Acid Production

**DOI:** 10.1002/fsn3.70612

**Published:** 2025-07-14

**Authors:** Melike Demirkol, Zekai Tarakçi

**Affiliations:** ^1^ Department of Food Engineering, Faculty of Engineering and Architecture Tokat Gaziosmanpaşa University Tokat Türkiye; ^2^ Department of Food Engineering, Faculty of Agriculture Ordu University Ordu Türkiye

**Keywords:** dairy products, dietary fiber, fruit by‐products, in vitro fermentation

## Abstract

This study evaluated the potential of fruit juice processing wastes as a prebiotic source and functional food ingredient in probiotic yogurt production. Here, it was aimed to demonstrate the prebiotic effect of apricot, peach, apple, and grape pomace from fruit juice industry waste in yogurt in vitro by determining the composition through microbial short‐chain fatty acids (SCFA) using gas chromatography. In addition, the effect of these pomaces on the quality characteristics of yogurt was investigated. During microbial fermentation in yogurt samples to which each fruit pomace was added at different ratios, there were gradual significant increases in the amounts of acetate, butyrate, and propionate, which are known as short‐chain fatty acids and have important beneficial effects for colonic microbiota, for all samples (*p* < 0.05). The main monosaccharide observed by monosaccharide analysis was glucose, followed by galactose. Microbiological analysis of the probiotic yogurt showed that the number of *Streptococcus thermophilus* and *Lactobacillus delbrueckii* ssp. *bulgaricus* did not change considerably during storage, and *Lactobacillus acidophilus* made better use of peach and apricot pomace as a prebiotic source. Based on the results of the sensory parameters, it can be said that the addition of 1% of apple, apricot, and peach grape pomace to yogurt is more liked by consumers. This study demonstrated that various fruit pomace fibers that can be easily applied to yogurt exhibited positive effects on gut microbiota by boosting SCFA. This study yielded significant findings about gut microbiota and prebiotic activity, contributing to both prebiotic research and the utilization of these components in the food sector.

## Graphical abstract

This study evaluated fruit juice processing wastes (apricot, peach, apple, and grape pomace) as prebiotic sources in probiotic yogurt. The addition of these pomaces led to significant increases (*p* < 0.05) in short‐chain fatty acids (acetate, butyrate, propionate), benefiting gut microbiota. Monosaccharide analysis identified glucose as the primary sugar. Microbiological analysis showed stable probiotic bacteria counts, with *Lactobacillus acidophilus* utilizing peach and apricot pomace effectively. Sensory evaluation favored 1% apple, apricot, and peach pomace. The findings support fruit pomace as a functional food ingredient.

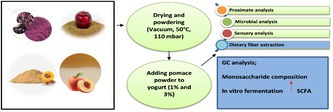

## 1 Introduction

Dietary fibers are carbohydrates that are not broken down in the upper digestive tract of the human body when consumed, but reach the colon, where they are utilized as energy sources by the colonic microflora. These components provide positive health effects due to their ability to regulate colonic microflora (Hamaker and Tuncil 2014; Koropatkin et al. 2012). The composition of gut microbiota is strongly influenced by host diet, and selective consumption of dietary fibers has been shown to regulate microbial diversity and species balance in the colon (Zhuang et al. 2024). This effect is based on the relationship between the chemical structures and binding types of dietary fibers and the genetic content of intestinal microorganisms. The ability of microorganisms to metabolize specific dietary fibers is influenced by their genetic composition. Extensive research, both in vitro and in vivo, has been conducted to investigate the effects of dietary fibers on colonic microbiota (Davis et al. 2010). These studies aim to identify dietary fibers that promote the proliferation of beneficial bacterial species such as Bifidobacteria and Lactobacilli while enhancing the production of short‐chain fatty acids (SCFAs) (Mazhar et al. 2023). Optimizing SCFA production is essential, as these gut microbiota‐derived metabolites serve as energy sources for colonocytes, regulate immune and inflammatory responses, and contribute to maintaining gastrointestinal health (Archana et al. 2024).

Cereal‐based dietary fibers (e.g., from breakfast cereals, bakery products, biscuits, etc.) are widely promoted and consumed. However, in the last decade, high dietary fiber ingredients derived from fruits such as citrus fruits and apples have been introduced to the market. Fruit dietary fibers are recognized for their superior nutritional quality compared to cereal fibers. They possess a balanced composition, with a higher total fiber content, an ideal soluble‐to‐insoluble dietary fiber ratio, excellent water and fat retention capacities, and lower energy value and phytic acid levels. Additionally, they contain essential bioactive compounds (Larrauri 1999). Studies indicate that soluble fibers in fruits and vegetables are highly fermentable and contribute significantly to intestinal fermentation (Chen et al. 2022; Ge et al. 2022).

With growing public interest in the health benefits of probiotics, numerous products have been developed as carrier foods to enhance health and nutrition (Jia et al. 2016). Yogurt, a widely consumed fermented dairy product with proven health benefits, is an ideal vehicle for incorporating various bioactive and functional compounds. However, such additions can alter the structure of yogurt gel during gastrointestinal digestion. Research examining their impact on the bioaccessibility of nutrients and bioactives often employs model foods like milk. Additionally, added ingredients can affect nutrient digestibility and significantly change the structural properties of the food (Fernandez and Marette 2017).

The nutritional value and technical applications of yogurt are primarily influenced by its components, including milk proteins, prebiotics, and plant‐based additives. Yogurt fortified with probiotics and prebiotics is associated with numerous health benefits, including improved immune and gastrointestinal function, enhanced lactose digestion, relief from inflammatory bowel conditions, and reduced allergic responses. In addition to these health benefits, consumer acceptance also depends heavily on the yogurt's appearance, texture, and flavor. Changes in processing or added components may affect texture, acidity, starter culture activity, and storage stability (Oh et al. 2016).

Today, efforts to develop alternative food products that help increase daily fiber intake continue. The food industry generates a significant amount of solid and liquid waste during production, processing, and consumption. Managing and recycling these wastes is increasingly important due to environmental concerns and the loss of valuable nutrients (Laufenberg et al. 2003).

Recent studies have focused on discovering plant‐based prebiotics, although limited research has been conducted on the functional and prebiotic properties of plant extracts in yogurt. Plant extracts have demonstrated antibacterial, antioxidant, and prebiotic potential. Therefore, developing novel plant‐derived prebiotics that enhance the growth of lactic acid bacteria is of growing interest. Fruit juice processing waste offers a potential source of such prebiotics for yogurt production. Accordingly, this research provides valuable insight into the application of fruit waste‐derived dietary fibers in the food industry. This study aimed to investigate the usability of waste products from the fruit juice processing industry (apple, peach, apricot, and grape pomaces) in the production of functional foods.

In our previous work, we examined the effects of these dietary fibers on monosaccharide composition and short‐chain fatty acid production through in vitro colonic microbial fermentation (Demirkol 2021). In this part of the study, the fruit pomaces were added to yogurt as sources of dietary fiber and potential prebiotic ingredients. We evaluated their effects on the physicochemical, sensory, and microbiological characteristics of yogurt during storage, as well as their impact on SCFA production and monosaccharide composition through fermentation by colonic microbiota.

## 2 Material and Methods

### 2.1 Preparation of Fruit Wastes

The pomaces of apricot ( *Prunus armeniaca* ), peach ( *Prunus persica* ), grape ( *Vitis vinifera* ), and apple ( *Malus domestica* ) used in the study were obtained from the Dimes Fruit Juice Factory located in Tokat, Turkey. Peach and apricot pomaces were collected in July 2019, while grape and apple pomaces were obtained in September 2019. Subsequently, the fruit pomace samples were dried in a vacuum oven at 55°C and 110 mbar pressure until a constant weight was achieved. After drying, the samples were further reduced in particle size by passing them through a coffee grinder and stored in sealed bags at −18°C until they were analyzed.

### 2.2 Proximate Analysis of Fruit Wastes

Information on the proportion of water soluble and insoluble dietary fiber from basic analysis of fruit waste and in vitro digestion analysis given elsewhere (Demirkol 2021).

### 2.3 Functional Properties of Fruit Wastes

To determine solubility, 0.5 g of pomace samples were weighed into falcon tubes, followed by the addition of 50 mL of distilled water. The mixture was homogenized using a vortex for 2 min and then centrifuged at 3000 ***g*** for 5 min. After centrifugation, 25 mL of the supernatant was subjected to drying at 105°C for 5 h. The solubility percentage was determined based on the weight difference (de Moraes Crizel et al. 2013). To measure water‐holding and oil‐holding capacities, 0.5 g of fruit pomace samples were weighed into falcon tubes, and 25 mL of distilled water and 10 mL of olive oil were added. The mixture was homogenized with a vortex and left at room temperature for 1 h. Then, falcon tubes were centrifuged at 1500 ***g*** for 10 min. The remaining solid portion was weighed after removing the water and oil phases. The absorbed amount of oil and water was determined in g on a dry matter basis. To analyze the swelling capacity, 0.5 g of sample was mixed with 10 mL of distilled water. The mixture was homogenized with a vortex and allowed to stand at room temperature for 18 h. As a result, the volume of the swollen pomace was recorded in milliliters on a dry matter basis (Gouw et al. 2017; Hassan et al. 2011).

### 2.4 Yogurt Production

The study was planned to produce set‐type probiotic yogurt using wastes obtained from fruit juice processing, including apple, apricot, peach, and grape pomace. The fruit pomace was incorporated into yogurt samples at concentrations of 1% and 3% based on both the results of our previous study (Demirkol and Tarakci 2018), which included quality analyses and sensory evaluations, and the preliminary trials conducted within the scope of this study. Pasteurized milk used for yogurt production (MİS Süt, Ak Gıda San. ve Tic A.Ş., Sakarya, Turkey, Carbohydrate: 4.7%, Fat: 3.1%, Protein: 2.8%) was obtained from a local market. The milk was pasteurized at 85°C for 20 min before being cooled to 43°C–45°C. It was then mixed with culture and pomace and fermented at 43°C until it reached a pH of 4.6. Finally, the yogurt was stored at 4°C overnight. The yogurt production process incorporated a traditional yogurt culture, YC‐350 (Chr. Hansen (Denmark), comprising *Streptococcus thermophilus* and *Lactobacillus delbrueckii* subsp. *bulgaricus* in a 1:1 ratio, employing a Direct Vat Set culture at a rate of 15 g/500 kg of milk), in combination with *Lactobacillus acidophilus* La5 (Chr. Hansen (Denmark), utilizing a Direct Vat Set culture at a rate of 25 g/250 kg of milk, corresponding to 10^7^ colony‐forming units per gram), as the probiotic culture. Analyses were performed on duplicate samples on days 1, 7, 14, and 21.

### 2.5 Proximate Analysis of Yogurts

The pH value of the yogurts was analyzed using a pH meter (inoLab, Weilheim, Germany) over a 21‐day storage period. To evaluate the titratable acidity, 9 g of yogurt was combined with 9 mL of distilled water and titrated to pH 8.2 using a 0.1 N NaOH (International, A 2000).

To determine the dry matter content, approximately 3–4 g of the sample was dried at 105°C until reaching a constant weight. The percentage of dry matter was then calculated based on the weight loss.

Konica Minolta CR‐5 (Japan) color meter was used to assess the color characteristics of yogurt. Readings were taken from five different regions of each sample, and the color values L, a, and b were recorded.

Viscosity measurements were conducted using a sine wave‐type pulsating viscometer (SV‐10, A&D Inc., Japan), which operates on the principle of the electric current needed to achieve resonance between two sensor plates at a constant frequency. Measurements were performed in duplicate at 10°C. Before measurement, the samples were stirred clockwise for 40 s and transferred to the sample container of the device in a volume of approximately 40 mL. Viscosity was determined in cP.

To test whey separation in yogurt, 10 g of the sample was centrifuged at 800 ***g*** for 10 min at 4°C. The serum content was then determined and presented as a percentage (Demirkol and Tarakci 2018).

### 2.6 Microbial Analysis of Yogurts

Microbiological analyses were conducted on days 1, 7, 14, and 21 of storage. Colony counts of *Lactobacillus acidophilus* La5 were performed using the Geraldi et al. (2018) method. Colonies that developed on de Man, Rogosa, and Sharpe (MRS) agar with 0.5 mg/L clindamycin (TCI, Tokyo) under anaerobic conditions at 37°C for 48–72 h were then counted.

*Lactobacillus delbrueckii* subsp. *bulgaricus* was determined by counting colonies at 37°C for 48 h in anaerobic conditions (Genbox Jar, anaerobic indicator) on MRS agar adjusted to pH 5.2 with acetic acid. *Streptococcus thermophilus* was determined by counting colonies on M17 agar medium at 37°C for 48 h (Chouchouli et al. 2013). Yeast and mold were determined by counting the colonies that developed after 4 days at 25°C using YGC agar medium.

### 2.7 Dietary Fiber Extraction of Yogurts

Dietary fibers were extracted from yogurt samples using an in vitro upper gastrointestinal digestion protocol (Tuncil et al. 2018). Lyophilized yogurt was mixed with phosphate buffer (84 mL per 12 g of sample) and adjusted to 37°C. The pH was set to 2.5 using 6 M HCl, and pepsin (2 mL per 12 g) was added, followed by a 30 min incubation at 37°C with continuous stirring. The pH was then raised to 6.9 with 6 M NaOH, and pancreatin (4 mL per 12 g) was added, with a subsequent 90 min incubation under the same conditions. Heating at 85°C for 20 min deactivated the enzymes. The samples were dialyzed for 36 h in deionized water using a 3500 Da molecular weight cut‐off tube, with water changes every 12 h. Finally, the lyophilized samples were analyzed for monosaccharide composition, with extraction performed on both 3% pomace and control samples.

### 2.8 Neutral Sugar Composition

Monosaccharide types and quantities in yoğurt dietary fibers were determined using gas chromatography (GC; Shimadzu GC‐2030) with an SP2330 column, based on the alditol acetate derivatization method of Pettolino et al. (2012). Detailed GC conditions were adapted from Tuncil et al. (2016). Samples were injected using an autosampler (Shimadzu AOC‐20i) into a gas chromatograph with a Restek Rtx‐2330 column (30 m × 0.25 mm ID × 0.2 μm film thickness) and a flame ionization detector (GC‐FID 7890A; Agilent Technologies Inc.).

The operating parameters were as follows: injection temperature, 240°C; injection volume, 1 μL; split ratio, 1:10; helium carrier gas at 1.14 mL/min; detector temperature, 250°C; and a column temperature program with gradients: 160°C (7.15 min), ramped to 220°C at 4°C/min (4.10 min), to 240°C at 2.9°C/min (5.15 min), and to 260°C at 10.8°C/min (5.10 min). Pure sugars (rhamnose, mannose, xylose, arabinose, galactose, fucose, and glucose) served as standards, with all samples derivatized to alditol acetates before analysis. Each sample was analyzed in duplicate.

### 2.9 Determination of Uronic Acid (Acidic Monosaccharides)

Acidic monosaccharides (galacturonic acid and glucuronic acid) in yogurt dietary fibers were quantified using the AACC 32–25.01 Uppsala method (Association of Analytical Cereal Chemists (AACC) 2000). Dietary fiber samples (50 mg) were treated with 0.3 mL of 12 M sulfuric acid and incubated at 30°C for 1 h. Then, 8.4 mL of distilled water was added, and the mixture was autoclaved at 125°C for 1 h. After cooling, 100 μL of the filtrate was combined with 100 μL of a sodium chloride/boric acid solution and 1.6 mL of 18 M sulfuric acid, followed by incubation at 70°C for 40 min. After adding 80 μL of a 3,5‐dimethylphenol solution, the absorbance was detected with a spectrophotometer at 400 and 450 nm. A standard curve was created using a galacturonic acid monohydrate standard solution, and the uronic acid content was determined as a percentage of the weight. Each sample was analyzed in duplicate.

### 2.10 In Vitro Fermentation

Short‐chain fatty acids in dietary fibers extracted from yogurt samples were determined under in vitro conditions using stool microflora obtained from healthy humans, following the method outlined by Sayar et al. (2007). For in vitro fermentation, fecal samples were collected from three healthy omnivorous individuals (two females aged 30 and 36, and one male aged 35) who had not used antibiotics for at least 3 months. For each time point (0, 6, 12, and 24 h), 50 mg of dietary fiber was placed into fermentation tubes. The fiber samples were mixed with 4 mL of an anaerobic fermentation media containing 3.7 g Brain Heart Infusion Broth without Dextrose (BHI), 93 mL distilled water, 2 mL L‐cysteine hydrochloride (12.5 g/L), 5 mL Na_2_CO_3_ (80 g/L), and 0.1 mL resazurin (1 g/L). Fecal samples from three donors were individually homogenized with BHI broth at a 1:3 (w/v) ratio and filtered through four layers of cheesecloth. Equal volumes of the filtered samples were then pooled to obtain a uniform fecal slurry. From this mixture, 1 mL was added to each fermentation tube. The tubes were sealed with rubber stoppers and incubated anaerobically (80% N_2_, 20% CO_2_) at 37°C in a shaking incubator, set to 120 rpm, for 24 h. After incubation, 1 mL from each tube was collected for short‐chain fatty acid analysis. Lactulose, a quickly fermentable compound, was utilized as a positive control. The Ordu University Clinical Research Ethics Committee approved this study's analysis to ensure that it complied with ethical principles and guidelines (code: 2019/115). Written informed consent was obtained from all donors prior to fecal sample collection.

### 2.11 SCFA Measurements

Short‐chain fatty acids (SCFAs) have been obtained from fecal samples using a method by Tuncil et al. (2017) and analyzed via gas chromatography (Shimadzu GC‐2030). An internal standard mixture of 4‐methylvaleric acid, copper sulfate pentahydrate, phosphoric acid, and distilled water was prepared, and 100 μL of this mixture was added to the samples, which were stored at −20°C until analysis. Samples were thawed and centrifuged, and then 4 μL of the supernatant was injected into a gas chromatograph equipped with a Stabilwax‐DA silica capillary column and a flame ionization detector (GC‐FID 7890A; Agilent Technologies Inc.) using a split ratio of 1:50. The injector temperature was set to 230°C, and the initial oven temperature was 100°C. The oven temperature was programmed to increase at a rate of 8°C/min to 200°C, where it was held for 3 min. Helium was used as the carrier gas at a flow rate of 0.75 mL/min; FID temperature at 250°C. SCFA concentrations were quantified by comparing peak areas to the internal standard, 4‐methylvaleric acid.

### 2.12 Sensory Analyses

The sensory evaluation of yogurts with fruit waste powder additives was conducted by a panel of 10 individuals, all academic staff members from Ordu University, Faculty of Agriculture, Department of Food Engineering, using a sensory evaluation scorecard. The panelists were requested to assess the sensory attributes of the yogurt samples using a 10‐point hedonic scale, covering categories such as appearance, texture, taste, odor, and overall acceptability.

### 2.13 Statistical Analysis

The results from the evaluations were statistically analyzed using analysis of variance (ANOVA) with SPSS 20 software. Tukey's post hoc test was employed to detect significant variances between groups, with an alpha level of 0.05. All results represent the mean of two replicates.

## 3 Results and Discussion

### 3.1 Functional Properties of Dried Fruit Pomace

As shown in Table 1, the swelling capacities (SC) of the pomaces ranged between 4.28 and 8.44. Peach pomace exhibited the highest SC, while grape and apricot pomaces had the lowest values (*p* < 0.05). Water holding capacity (WHC) varied from 3.22 to 7.08, with peach pomace again showing the highest WHC and grape pomace the lowest (*p* < 0.05). In contrast, Figuerola et al. (2005) reported lower WHC values (1.62–2.26) for fruit juice processing by‐products such as grapefruit, apple pomace, citrus, and orange peel. In that study, the WHC of apple wastes was found to be lower than that of grape wastes, which differs from our results. The variation in WHC may be attributed to the presence of highly soluble fibrous materials (Hassan et al. 2011). The WHC of dietary fibers (DF) reflects their pore volume and provides insight into their behavior during intestinal transit. Fibers with high WHC can increase chewing time, promote satiety, and enhance fecal bulk (Lv et al. 2017). Therefore, peach pomace, with its high water retention capacity compared to other pomaces in the study, has the potential to be used as a functional ingredient to improve food quality and support body weight management.

**TABLE 1:** Functional properties of dried fruit pomaces used in yogurt fortification.

Components	Content (dry weight basis)			
	Apple pomace	Apricot pomace	Peach pomace	Grape pomace
SC (mL/g DM)	6.41 ± 0.00^C^	6.48 ± 0.01^B^	8.44 ± 0.02^A^	4.28 ± 0.00^D^
WHC (g water/g DM)	5.23 ± 0.26^B^	4.30 ± 0.06^bc^	7.08 ± 0.59^A^	3.22 ± 0.09^C^
OHC (g oil/g DM)	2.08 ± 0.37^A^	2.84 ± 0.18^A^	2.81 ± 0.15^A^	2.78 ± 0.02^A^
Solubility (%, 25°C)	18.92 ± 1.44^D^	37.33 ± 0.33^B^	39.58 ± 0.31^A^	30.5 ± 0.14^C^

*Note:* ^A–D^Values with different letters in the same row differ from one another at the *p* < 0.05 level.

Abbreviations: OHC, Oil holding capacity; SC, Swelling capacity; WHC, Water holding capacity.

As shown in Table 1, the oil holding capacity (OHC) of fruit pomaces varied between 2.08 and 2.84 (g/g). There were no significant differences in OHC among the pomace types (*p* > 0.05). Seker et al. (2009) reported water holding capacities of 6.6 and 6.7 g/g for apple and apricot pomaces, respectively. In another study, Martinez et al. (2012) found that the OHC of dietary fibers obtained from various fruit by‐products ranged between 0.7 and 1.6 g/g. Cui et al. (2017) reported OHC, WHC, and SC values of 1.7 g/g, 4.2 g/g, and 3.2 mL/g for apple pomace, and 2.3 g/g, 6.4 g/g, and 6.5 mL/g for grape pomace, respectively, in a study examining dietary fibers from fruits and vegetables. The findings of this study were lower than the findings of our study in apple pomace and higher than the findings of our study in grape pomace. These differences can be attributed to the structure and content of the cell wall material, differences in particle size and porosity, and environmental conditions like pH and temperature (Lario et al. 2004).

As presented in Table 1, the solubility of fruit pomaces ranges between 18.92% and 39.58%. Peach pomace exhibited the highest solubility, while apple pomace showed the lowest (*p* < 0.05). de Moraes Crizel et al. (2013) reported solubility values of 28.95% and 28.90% for orange pomace and orange peel fibers, respectively. Compared to these values, peach pomace in this study demonstrated a higher soluble fraction. Moreover, it appears to have a favorable balance between soluble and insoluble fibers. This balanced fiber composition, along with the rich phytochemical content of fruit and vegetable pomace, can enhance hydration properties and fermentability (Sahni and Shere 2018). Additionally, studies have shown that the particle size and fiber composition of powders can influence their hydration and emulsification capacities (Calabuig‐Jiménez et al. 2022).

### 3.2 Physicochemical Analyses of Yogurts

**FIGURE 1:**
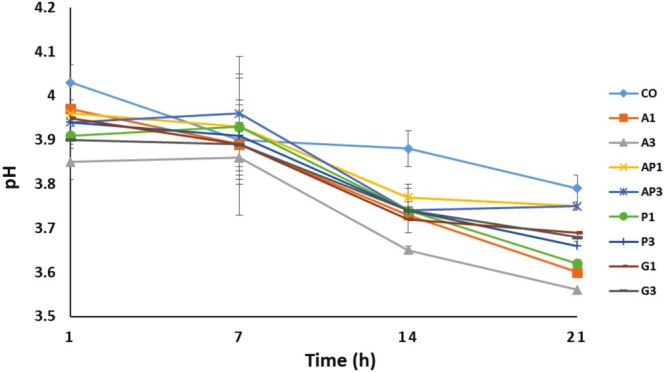
pH values of yogurt samples during storage. CO, control sample without pomace addition. A1, A3: yogurt with 1% and 3% apple pomace; AP1, AP3: 1% and 3% apricot pomace; P1, P3: 1% and 3% peach pomace; G1, G3: 1% and 3% grape pomace. Values represent means ± standard deviation.

The dry matter content of probiotic yogurts containing fruit pomace ranged from 12.33% to 14.59%. As shown in Figure 1, the pH values of the samples varied between 3.60 and 4.03. Overall, the pH of the yogurt samples tended to decrease during storage (*p* < 0.05). Although most samples showed a significant drop in pH (*p* < 0.05), the yogurt containing apricot pomace showed no statistically significant change by day 21 (*p* > 0.05). There was not a substantial variance in pH values between the samples at the initial stage of storage (*p* > 0.05), but significant variances were noticed at the final stage of storage (*p* < 0.05). Neither the pomace type nor its concentration had a significant effect on pH values on the initial day of storage (*p* > 0.05). The decrease in pH at the end of storage may be attributed to the natural acids present in the fruit pomaces. Helal et al. (2018) stated that the addition and percentage of inulin did not significantly influence yogurt pH. Geraldi et al. (2018) found no substantial pH changes during storage in yogurt enriched with jucara pulp. Srisuvor et al. (2013) also noted that polydextrose and inulin had no effect on the pH, syneresis, or titratable acidity of low‐fat yogurt. In the present study, no notable pH changes were observed until day 14. After that point, a general decline in pH levels became evident (*p* < 0.05).

**FIGURE 2:**
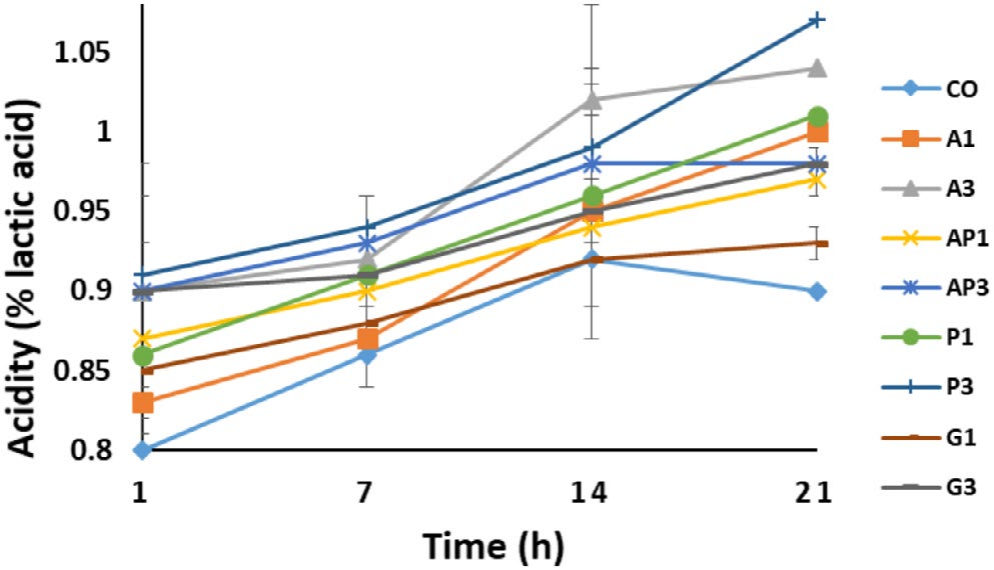
Titratable acidity (%) of yogurt samples during storage. CO, control sample without pomace addition. A1, A3: yogurt with 1% and 3% apple pomace; AP1, AP3: 1% and 3% apricot pomace; P1, P3: 1% and 3% peach pomace; G1, G3: 1% and 3% grape pomace. Values represent means ± standard deviation.

Figure 2 shows the titratable acidity of the yogurt samples, which ranged from 0.80% to 1.07%. At the start of the storage duration, there were no noteworthy variations in acidity among the samples (*p* > 0.05), indicating that the type and concentration of pomace had no initial impact. However, by the end of the storage period, acidity levels had increased significantly (*p* < 0.05). Tarakçı and Küçüköner (2003) similarly reported that the titratable acidity of fruit‐flavored yogurts increased during storage.

Color values of yogurts are presented in Table 2. Increasing the fruit pomace content slightly reduced the *L* values, although this decrease was not statistically significant (*p* > 0.05). A noticeable reduction in *L* value was observed in sample G3 at the beginning of storage (*p* < 0.05). No significant changes in *L* values were detected over the course of storage (*p* > 0.05). Marand et al. (2020) also reported that the L* values of yogurts remained stable during storage and noted a decrease in brightness with increasing linseed powder content.

**TABLE 2:** Color parameters of yogurt samples with fruit pomace during storage.

Parameter	Sample	Storage day			
		1	7	14	21
*L*	CO	79.65 ± 2.60^Aa^	77.99 ± 0.06^Aa^	77.05 ± 0.02^Aa^	76.59 ± 0.65^Aa^
	A1	70.02 ± 5.96^Aab^	68.57 ± 0.40^Ab^	67.59 ± 0.62^Ac^	68.49 ± 0.07^Ac^
	A3	62.70 ± 7.54^Ab^	60.61 ± 1.43^Ad^	60.00 ± 0.87^Ae^	61.25 ± 0.05^Af^
	AP1	71.53 ± 5.11^Aab^	67.81 ± 2.08^Ab^	68.28 ± 1.04^Ac^	68.42 ± 0.07^Ac^
	AP3	64.33 ± 6.14^Ab^	60.19 ± 2.38^Ad^	60.77 ± 0.79^Ae^	62.20 ± 0.13^Ae^
	P1	72.08 ± 4.63^Aab^	70.18 ± 1.52^Ab^	70.19 ± 0.41^Ab^	71.83 ± 0.10^Ab^
	P3	66.82 ± 6.55^Aab^	64.09 ± 1.31^Ac^	64.31 ± 0.51^Ad^	65.27 ± 0.04^Ad^
	G1	60.92 ± 4.31^Abc^	56.79 ± 0.70^Ae^	57.05 ± 0.02^Af^	58.55 ± 0.07^Ag^
	G3	49.14 ± 5.99^Ac^	46.72 ± 0.65^Af^	46.86 ± 1.00^Ag^	48.21 ± 0.18^Ah^
a	CO	−2.59 ± 0.68^Ae^	−2.79 ± 0.07^Ag^	−2.8 ± 0.18^Ah^	−2.83 ± 0.05^Ah^
	A1	0.55 ± 0.10^Ade^	0.52 ± 0.06^Ae^	0.44 ± 0.07^Af^	0.58 ± 0.02^Af^
	A3	2.07 ± 0.27^Ac^	1.78 ± 0.21^Ac^	1.68 ± 0.16^Ad^	1.96 ± 0.03^Ac^
	AP1	0.31 ± 0.05^Ade^	0.41 ± 0.13^Ae^	0.26 ± 0.07^Af^	0.53 ± 0.01^Af^
	AP3	2.06 ± 0.13^Ac^	1.88 ± 0.23^Ac^	2.01 ± 0.08^Ac^	1.89 ± 0.02^Ad^
	P1	−0.26 ± 0.11^Ae^	−0.36 ± 0.24^Af^	−0.25 ± 0.08^Ag^	−0.1 ± 0.02^Ag^
	P3	1.12 ± 0.07^Acd^	1.22 ± 0.07^Ad^	1.21 ± 0.04^Ae^	1.17 ± 0.01^Ae^
	G1	4.22 ± 0.66^Ab^	4.00 ± 0.12^Ab^	4.16 ± 0.00^Ab^	4.08 ± 0.02^Ab^
	G3	6.10 ± 0.91^Aa^	5.53 ± 0.07^Aa^	5.82 ± 0.06^Aa^	5.96 ± 0.02^Aa^
b	CO	6.84 ± 0.20^Ad^	6.15 ± 0.04^Ae^	5.92 ± 0.25^Ae^	6.30 ± 0.03^Af^
	A1	7.90 ± 0.77^Acd^	7.51 ± 0.08^Ad^	7.26 ± 0.14^Ad^	7.26 ± 0.01^Ae^
	A3	10.13 ± 0.95^Abc^	8.80 ± 0.22^Ac^	8.61 ± 0.13^Ac^	8.70 ± 0.02^Ac^
	AP1	9.24 ± 1.50^Abc^	10.03 ± 0.57^Ab^	9.85 ± 0.04^Ab^	9.64 ± 0.03^Ab^
	AP3	14.31 ± 1.38^Aa^	12.34 ± 0.23^ABa^	12.39 ± 0.07^ABa^	11.95 ± 0.03^Ba^
	P1	8.91 ± 0.86^Acd^	8.43 ± 0.27^Ac^	8.46 ± 0.19^Ac^	8.46 ± 0.03^Ad^
	P3	11.24 ± 1.11^Ab^	9.95 ± 0.07^Ab^	9.91 ± 0.16^Ab^	9.67 ± 0.01^Ab^
	G1	−0.51 ± 0.41^Ae^	−0.31 ± 0.05^Af^	−0.30 ± 0.09^Af^	−0.18 ± 0.02^Ag^
	G3	−1.57 ± 0.29^Ae^	−1.28 ± 0.17^Bg^	−1.42 ± 0.25^Bg^	−1.30 ± 0.02^ABh^

*Note:* ^A‐B^Values with different letters in the same row differ from one another at *p* < 0.05 level. ^a–h^Values with different letters in the same column differ from one another at *p* < 0.05 level. A1; 1% apple pomace. A3; 3% apple pomace. AP1; 1% apricot pomace. AP3; 3% apricot pomace. P1; 1% peach pomace. P3; 3% peach pomace. G1; 1% grape pomace. G3; 3% grape pomace.

Abbreviation: CO, control sample.

As shown in Table 2, yogurts enriched with grape pomace had the highest a values on the primary day of storage (*p* < 0.05). Although a values did not change significantly during storage (*p* > 0.05), increasing the pomace concentration led to higher a values overall (*p* < 0.05). At both the beginning and end of storage, the highest b value was observed in the AP3 sample (*p* < 0.05), while the lowest was found in samples containing grape pomace (*p* < 0.05). Except for the AP3 and G3 samples, no significant changes in b values were detected during the storage period (*p* > 0.05).

Table 3 indicates that the amount of whey separation in the samples varied between 25.87% and 32.53%. These results are consistent with those reported by Tseng and Zhao (2013), who observed serum separation between 17.25% and 33.58% in yogurts to which powdered pomace was added after fermentation. These values coincide with the values in our study. In general, neither the type nor the concentration of pomace significantly affected the extent of whey separation (*p* > 0.05). A previous study found that syneresis in yogurt with added grape pomace decreased compared to the control. Similarly, in our study, syneresis was reduced. This decrease may be due to the pH drop during storage, which causes gel contraction and increases gel strength (Demirkol and Tarakci 2018).

**TABLE 3:** Syneresis (%) in yogurt samples throughout storage.

Sample	Storage day			
	1	7	14	21
CO	31.94 ± 4.13^Aa^	28.22 ± 0.98^ABa^	28.21 ± 1.94^ABa^	25.87 ± 2.22^Bb^
A1	31.43 ± 3.48^Aa^	30.56 ± 3.11^Aa^	31.66 ± 2.67^Aa^	32.53 ± 0.65^Aa^
A3	30.00 ± 1.85^Aa^	30.63 ± 3.41^Aa^	29.66 ± 0.13^Aa^	28.34 ± 2.38^Aab^
AP1	30.34 ± 2.75^Aa^	28.89 ± 0.01^Aa^	26.70 ± 2.18^Aa^	30.57 ± 0.01^Aab^
AP3	29.23 ± 1.17^Aa^	30.36 ± 1.62^Aa^	29.09 ± 7.03^Aa^	29.66 ± 4.89^Aab^
P1	30.46 ± 0.97^Aa^	30.02 ± 3.17^Aa^	30.11 ± 3.82^Aa^	30.38 ± 1.19^Aab^
P3	27.87 ± 2.36^Aa^	28.77 ± 0.03^Aa^	27.67 ± 4.65^Aa^	29.15 ± 3.38^Aab^
G1	29.32 ± 3.88^Aa^	26.99 ± 1.49^Aa^	27.42 ± 2.67^Aa^	28.18 ± 3.25^Aab^
G3	29.96 ± 2.72^Aa^	28.90 ± 2.52^Aa^	27.68 ± 1.44^Aa^	29.82 ± 0.38^Aab^

*Note:* ^A‐B^Values with different letters in the same row differ from one another at *p* < 0.05 level. ^a‐b^Values with different letters in the same column differ from one another at *p* < 0.05 level. A1; 1% apple pomace. A3; 3% apple pomace. AP1; 1% apricot pomace. AP3; 3% apricot pomace. P1; 1% peach pomace. P3; 3% peach pomace. G1; 1% grape pomace. G3; 3% grape pomace.

As shown in Table 4, viscosity values of the yogurt samples decreased with increasing pomace content (*p* < 0.05). Similar findings were reported by Paseephol et al. (2008), who observed that inulin‐supplemented yogurt had lower apparent viscosity than non‐supplemented samples. Likewise, Sah et al. (2016) found that yogurt fortified with pineapple peel powder showed significantly lower viscosity compared to control samples during storage. This reduction is likely due to the weakening of the yogurt gel structure at higher pomace concentrations, as also suggested by Lee and Lucey (2010). In general, viscosity values increased by the end of the storage period. Sendra et al. (2010) reported that the addition of orange by‐products increased yogurt viscosity and improved water absorption. Several studies have noted that this increase may result from structural improvements in the yogurt matrix during storage. These changes are likely associated with protein reorganization and enhanced protein–fiber interactions, which contribute to a stronger and thicker gel consistency (Isleten and Karagul‐Yuceer 2006; Lee and Lucey 2010). Marand et al. (2020) also noted that the presence of fiber and protein compounds can promote viscous gel formation by enhancing water‐holding capacity.

**TABLE 4:** Viscosity (cP) of yogurt samples containing fruit pomace during storage.

Sample	Storage day			
	1	7	14	21
CO	266.96 ± 15.56^Ba^	275.63 ± 16.4^Ba^	261.24 ± 45.65^Ba^	376.79 ± 25.08^Aa^
A1	226.09 ± 3.36^Ab^	157.46 ± 17.33^Ab^	227.16 ± 46.85^Aab^	264.23 ± 4.08^Aab^
A3	89.350 ± 1.45^Bd^	137.19 ± 29.48^Abcd^	132.12 ± 21.38^Aab^	160.02 ± 55.15^Abc^
AP1	144.47 ± 3.41^Ac^	148.97 ± 12.18^Abc^	143.22 ± 8.65^Aab^	162.32 ± 3.10^Abc^
AP3	101.10 ± 29.48^Ad^	120.69 ± 14.31^Abcde^	101.89 ± 3.92^Ab^	116.59 ± 13.73^Ac^
P1	83.370 ± 1.88^Bd^	88.66 ± 5.24^Be^	123.05 ± 32.45^Aab^	176.63 ± 49.24^Abc^
P3	119.33 ± 24.68^Acd^	92.79 ± 15.55^Ade^	92.66 ± 1.82^Ab^	105.66 ± 35.95^Ac^
G1	98.54 ± 16.14^Bd^	127.03 ± 24.75^Bbcde^	148.23 ± 24.19^Bab^	265.07 ± 30.56^Aab^
G3	101.87 ± 11.43^Bd^	106.12 ± 26.15^ABcde^	135.2 ± 29.42^ABab^	172.76 ± 27.29^Abc^

*Note:* ^A‐B^Values indicated with different letters in the same row differ from one another at *p* < 0.05 level. ^a–e^Values indicated with different letters in the same column differ from one another at *p* < 0.05 level. A1; 1% apple pomace. A3; 3% apple pomace. AP1; 1% apricot pomace. AP3; 3% apricot pomace. P1; 1% peach pomace. P3; 3% peach pomace. G1; 1% grape pomace. G3; 3% grape pomace.

### 3.3 Microbiological Analyses of Yogurt Samples

According to Table 5, *Lactobacillus bulgaricus* counts in probiotic yogurt samples enriched with fruit pomace ranged from 7.77 to 8.22 log cfu/g. In all samples, these values were slightly lower than those of *Streptococcus thermophilus* (*p* > 0.05). This observation is consistent with previous studies. Abdel‐Hamid et al. (2020) reported lower Lactobacilli counts compared to Streptococci. Similarly, Sah et al. (2015) found Lactobacilli counts between 7.31 and 8.28 log cfu/g, while Göçer et al. (2016) reported values ranging from 7.8 to 8.6 log cfu/g.

**TABLE 5:** Microbiological counts (log cfu/g) of probiotic bacteria in yogurt samples with fruit pomace.

	Storage day				
	Sample	1	7	14	21
*Lactobacillus bulgaricus*	CO	8.08 ± 0.28^Aa^	8.09 ± 0.12^Aa^	8.22 ± 0.14^Aa^	7.77 ± 0.10^ABc^
	A1	7.92 ± 0.16^Aa^	8.07 ± 0.13^Aa^	8.10 ± 0.03^Aa^	8.02 ± 0.03^Aab^
	A3	7.93 ± 0.02^Aa^	7.86 ± 0.09^Aa^	7.91 ± 0.01^Aa^	7.94 ± 0.08^Abc^
	AP1	8.05 ± 0.11^Aa^	8.03 ± 0.11^Aa^	8.01 ± 0.07^Aa^	8.18 ± 0.08^Aa^
	AP3	7.85 ± 0.07^Aa^	7.84 ± 0.00^Aa^	7.93 ± 0.06^Aa^	7.88 ± 0.04^Abc^
	P1	7.78 ± 0.16^Aa^	8.05 ± 0.08^Aa^	8.01 ± 0.19^Aa^	8.05 ± 0.01^Aab^
	P3	8.02 ± 0.08^Aa^	7.86 ± 0.00^Aa^	7.89 ± 0.06^Aa^	7.84 ± 0.01^Abc^
	G1	7.97 ± 0.18^Aa^	8.15 ± 0.02^Aa^	8.08 ± 0.09^Aa^	7.92 ± 0.02^Abc^
	G3	8.12 ± 0.09^Aa^	8.05 ± 0.06^Aa^	8.12 ± 0.02^Aa^	8.02 ± 0.03^Aab^
*Streptococcus thermophilus*	CO	8.53 ± 0.22^Aa^	8.48 ± 0.09^Aa^	8.56 ± 0.08^Aa^	8.47 ± 0.01^Aa^
	A1	8.43 ± 0.24^Aa^	8.24 ± 0.03^Aa^	8.39 ± 0.23^Aa^	8.48 ± 0.09^Aa^
	A3	8.55 ± 0.03^Aa^	8.19 ± 0.43^Aa^	8.53 ± 0.21^Aa^	8.46 ± 0.00^Aa^
	AP1	8.50 ± 0.28^Aa^	8.40 ± 0.19^Aa^	8.43 ± 0.18^Aa^	8.44 ± 0.05^Aa^
	AP3	8.41 ± 0.02^Aa^	8.46 ± 0.06^Aa^	8.47 ± 0.22^Aa^	8.52 ± 0.03^Aa^
	P1	8.52 ± 0.30^Aa^	8.30 ± 0.46^Aa^	8.49 ± 0.13^Aa^	8.54 ± 0.11^Aa^
	P3	8.53 ± 0.05^Aa^	8.38 ± 0.27^Aa^	8.54 ± 0.06^Aa^	8.59 ± 0.05^Aa^
	G1	8.56 ± 0.13^Aa^	8.34 ± 0.42^Aa^	8.45 ± 0.20^Aa^	8.68 ± 0.09^Aa^
	G3	8.56 ± 0.11^Aa^	8.50 ± 0.32^Aa^	8.48 ± 0.15^Aa^	8.54 ± 0.01^Aa^
*Lactobacillus acidophilus* La5	CO	7.28 ± 0.20^Aa^	7.05 ± 0.22^Ab^	6.33 ± 0.63^ABb^	5.74 ± 0.37^Bd^
	A1	7.56 ± 0.16^Aa^	7.26 ± 0.14^Aab^	6.64 ± 0.57^ABab^	6.21 ± 0.29^Bbc^
	A3	7.60 ± 0.02^Aa^	7.47 ± 0.17^Aa^	7.26 ± 0.20^ABa^	6.86 ± 0.06^Bab^
	AP1	7.36 ± 0.34^Aa^	7.35 ± 0.16^Aab^	6.88 ± 0.34^ABab^	6.61 ± 0.01^Babc^
	AP3	7.54 ± 0.12^Aa^	7.41 ± 0.01^Aab^	7.32 ± 0.22^ABa^	7.10 ± 0.15^Ba^
	P1	7.56 ± 0.03^Aa^	7.40 ± 0.10^Aab^	7.23 ± 0.10^ABa^	6.74 ± 0.15^Bab^
	P3	7.57 ± 0.05^Aa^	7.63 ± 0.15^Aa^	7.48 ± 0.14^ABa^	7.16 ± 0.11^Ba^
	G1	7.46 ± 0.19^Aa^	7.31 ± 0.26^Aab^	6.33 ± 0.20^Bb^	6.30 ± 0.43^Bbc^
	G3	7.47 ± 0.02^Aa^	7.44 ± 0.05^Aa^	6.96 ± 0.31^Bab^	6.35 ± 0.07^Cbc^

*Note:* ^A–C^Values with different letters in the same row differ from one another at *p* < 0.05 level. ^a–d^Values with different letters in the same column differ from one another at *p* < 0.05 level. A1; 1% apple pomace. A3; 3% apple pomace. AP1; 1% apricot pomace. AP3; 3% apricot pomace. P1; 1% peach pomace. P3; 3% peach pomace. G1; 1% grape pomace. G3; 3% grape pomace.

After 21 days of storage, no significant changes were observed in *L. bulgaricus* counts across the pomace‐added samples (*p* > 0.05). However, a slight, non‐significant decrease was noted in the control sample by the end of storage (*p* > 0.05). The stability of *L. bulgaricus* in the pomace‐supplemented yogurts may be attributed to the prebiotic effects of the fruit pomace, which likely supported the growth and viability of Lactobacilli during fermentation and subsequent storage.

Table 5 shows that *Streptococcus thermophilus* counts in probiotic yogurt samples with added fruit pomace ranged from 8.19 to 8.68 log cfu/g. Overall, *S. thermophilus* counts were higher than *L. bulgaricus* counts throughout the study. Ranadheera et al. (2012) similarly reported that *S. thermophilus* was more stable than *L. bulgaricus* in probiotic yogurt made with goat milk during storage. Neither the type nor the concentration of pomace had a significant effect on *S. thermophilus* counts during the storage period (*p* > 0.05). In line with our findings, Mani‐López et al. (2014) observed that probiotic bacteria did not inhibit *S. thermophilus* viability, and its population remained stable over 14 days of storage.

As shown in Table 5, *Lactobacillus acidophilus* counts in yogurts with fruit pomace addition ranged from 5.74 to 7.60 log cfu/g. While *L. acidophilus* counts remained stable during the early storage days, a slight decrease was observed by day 14 (*p* > 0.05), with a more significant reduction in the G1 and G3 samples (*p* < 0.05). From day 7 onward, samples with higher pomace content tended to have higher *L. acidophilus* counts compared to those with lower pomace, though these differences were not statistically significant (*p* > 0.05).

At the end of storage, viable probiotic bacteria decreased in all samples (*p* < 0.05), but *L. acidophilus* counts remained above the therapeutic minimum threshold (10^6–10^7 log cfu/g) in all cases. Similar results were reported by Güler‐Akın et al. (2018) for probiotic yogurt with added cellulose fiber. The control sample showed the most pronounced decrease by the final day (*p* < 0.05). Mortazavian et al. (2006) noted that such decreases may result from competition between *L. acidophilus* and yogurt bacteria, with acidity being a key factor affecting *L. acidophilus* survival. Acidity increased during storage, and by the end, AP3 and P3 samples had significantly higher *L. acidophilus* counts than the control, A1, G1, and G3 samples (*p* < 0.05). This suggests that apple, apricot, and peach pomaces were better utilized as prebiotic sources by *L. acidophilus* compared to grape pomace. Previous studies have shown that fruit pomace and fibers can increase probiotic populations in yogurt (Geraldi et al. 2018; Sah et al. 2016; Santo et al. 2012; Shori 2013). The viability of *L. acidophilus* depends on factors such as food composition, inoculum size, fermentation time, pH, and prebiotic presence (Helland et al. 2004). Prebiotics support probiotic survival and colonization in the gut (Zhang et al. 2024; Xue et al. 2024). No yeast or mold contamination was detected throughout storage.

### 3.4 Monosaccharide Composition

**FIGURE 3:**
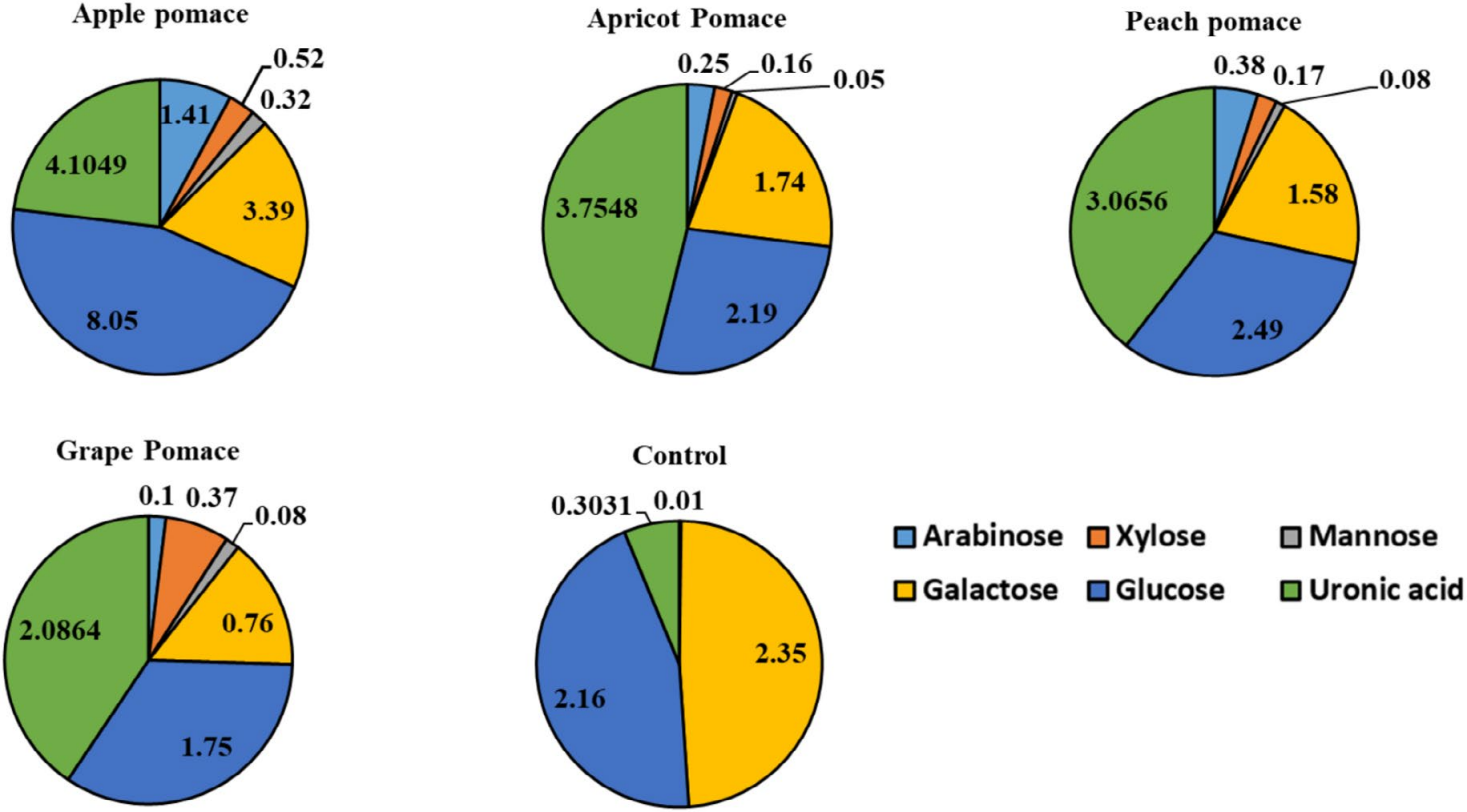
Monosaccharide profiles (%) of dietary fibers extracted from probiotic yogurt samples containing 3% fruit pomace. The control sample represents yogurt without pomace addition. Arabionse, xylose, mannose, galactose, glucose, and uronic acid levels were analyzed to assess fiber utilization and prebiotic potential.

Figure 3 presents the monosaccharide composition of yogurt dietary fibers, while Table S1 compares the fibers from yogurt and fruit pomace. Understanding the monosaccharide profile of dietary fibers from fruit pomace is important to evaluate their interactions with gut microbiota, the types of SCFAs produced, and their effects on gut health. Different bacterial species utilize specific monosaccharides, influencing microbial balance, fermentation rates, and production of beneficial metabolites. This knowledge supports the development of fiber‐based products aimed at improving digestion and gut health (Wang et al. 2015; Zeng et al. 2018). In this study, dietary fiber was extracted from probiotic yogurts containing 3% pomace. The monosaccharide composition of pomace fibers was previously reported by Demirkol (2021). Glucose was the predominant monosaccharide in yogurt fibers, followed by galactose (Figure 3). Apple pomace yogurt fibers contained the highest glucose and galactose levels, while grape pomace fibers had the lowest (*p* < 0.05). In earlier research, glucose was also the main monosaccharide in pomace fibers, followed by arabinose (Demirkol 2021). Peach pomace fibers had the highest arabinose, xylose, glucose, and mannose levels, which decreased significantly after fermentation in yogurt (*p* < 0.05). This suggests that yogurt bacteria utilized peach pomace as a prebiotic source, consuming these sugars. This aligns with microbiological data showing stable *Lactobacillus acidophilus* counts during storage in pomace‐supplemented yogurts. Yogurt fibers contained significantly less uronic acid than pomace fibers (*p* < 0.05) as shown in Table S1. Similar findings were reported by Xiong et al. (2023), who identified galactose and glucose as primary monosaccharides in yogurt. Li et al. (2020) found that yogurt polysaccharides mainly consist of galactose, glucose, mannose, and glucuronic acid. Lei et al. (2023) demonstrated that exopolysaccharides produced by *Lactobacillus plantarum* in yogurt include glucose, arabinose, rhamnose, mannose, and galactose. Tang et al. (2023) reported that *Lactobacillus delbrueckii* DMLD‐H1 can transport lactose, glucose, fructose, and mannose. They suggested that differences in monosaccharide composition among samples could be due to variations in fermentation strains.

### 3.5 Short‐Chain Fatty Acids During In Vitro Fermentation

Table 6 presents the amounts of short‐chain fatty acids (SCFAs) produced during in vitro fermentation. At the 6th hour, lactulose induced the highest acetate production (*p* < 0.05), followed by dietary fibers from apple pomace yogurt. The lowest acetate levels were found in fibers from grape pomace yogurt; however, differences among pomace fibers were not statistically significant (*p* > 0.05). Acetate production gradually increased throughout fermentation (*p* < 0.05). By the end of fermentation, yogurt with apple pomace produced significantly more acetate than those with apricot, peach, or grape pomace (*p* < 0.05). Similarly, the highest butyrate concentration was observed in apple pomace fibers, though other pomaces showed no significant differences (*p* > 0.05). At 24 h, propionate was highest in yogurts with apple and peach pomace, while grape and apricot pomace fibers showed lower levels. Overall, pomace fibers significantly enhanced SCFA production in yogurt (*p* < 0.05).

**TABLE 6:** Short chain fatty acid concentrations (mM) produced from yogurt dietary fibers during in vitro fermentation.

		Time (h)			
		0	6	12	24
Acetate	Apple		30.39 ± 0.44^Cb^	58.50 ± 2.98^Bb^	83.11 ± 3.62^Ab^
	Apricot		28.87 ± 2.83^Cb^	57.62 ± 1.09^Bb^	70.24 ± 10.11^Ac^
	Peach		27.84 ± 2.47^Cb^	58.65 ± 2.94^Bb^	77.98 ± 0.74^Ac^
	Grape		25.45 ± 0.04^Cbc^	53.23 ± 1.11^Bc^	74.57 ± 14.63^Ac^
	Control		25.86 ± 4.65^Bbc^	58.42 ± 0.45^Ab^	66.61 ± 1.86^Ad^
	Lactulose		57.86 ± 3.91^Ca^	85.48 ± 0.09^Ba^	101.22 ± 5.08^Aa^
	Blank	7.72 ± 0.05^D^	24.12 ± 5.09^Cbc^	55.90 ± 1.08^Bbc^	70.22 ± 2.11^Ac^
Butyrate	Apple		5.79 ± 0.07^Cb^	15.18 ± 0.88^Bb^	23.92 ± 1.19^Ab^
	Apricot		5.52 ± 0.65^Bbc^	15.26 ± 0.02^Ab^	19.82 ± 2.94^Ac^
	Peach		5.23 ± 0.54^Cbc^	14.67 ± 0.36^Bb^	21.5 ± 0.19^Abc^
	Grape		4.71 ± 0.16^Cc^	13.16 ± 0.74^Bc^	20.03 ± 4.37^Ac^
	Control		4.32 ± 0.64^Cc^	13.05 ± 0.02^Bc^	17.18 ± 0.52^Ad^
	Lactulose		7.56 ± 0.43^Ca^	20.05 ± 0.19^Ba^	25.66 ± 0.83^Aa^
	Blank	1.04 ± 0^D^	2.27 ± 0.7^Cd^	9.72 ± 0.03^Bd^	13.84 ± 0.76^Ae^
Propionate	Apple		9.65 ± 0.13^Cab^	20.39 ± 1.37^Ba^	27.27 ± 1.42^Aa^
	Apricot		9.06 ± 1.30^Bab^	20.60 ± 0.12^Aa^	23.33 ± 3.19^Ab^
	Peach		8.60 ± 0.42^Cbc^	19.69 ± 0.88^Bab^	25.47 ± 0.5^Aab^
	Grape		7.82 ± 0.16^Ccd^	17.8 ± 0.87^Bcd^	23.55 ± 4.83^Ab^
	Control		7.39 ± 1.08^Ccd^	18.71 ± 0.17^Bc^	21.7 ± 0.51^Ab^
	Lactulose		10.18 ± 0.61^Ca^	16.35 ± 0.02^Bcd^	21.99 ± 2.31^Ab^
	Blank	1.28 ± 0.02^D^	6.04 ± 1.38^Cd^	14.56 ± 0.54^Bd^	18.67 ± 0.7^Ac^

*Note:* ^A–C^Values indicated with different letters in the same row differ from one another at *p* < 0.05 level. ^a–e^Values indicated with different letters in the same column differ from one another at *p* < 0.05 level. TDF (SDF + IDF): mixture of water soluble and insoluble dietary fibers. Lactulose was used as a positive control. The blank does not contain any substrate.

Besides apple pomace, apricot and peach pomace also positively influenced probiotic microbial counts in yogurt. The lower SCFA production in grape pomace may be due to its higher content of insoluble fibers like cellulose, which are less fermentable by colonic bacteria. In contrast, apple pomace, rich in soluble fibers such as pectin, supports greater microbial fermentation and SCFA generation, particularly acetate. Various studies have shown that the source and composition of dietary fiber greatly affect its functional properties and behavior during intestinal transit (Ndeh et al. 2017; Cui et al. 2019; Demirkol 2021). In the control yogurt without pomace, SCFA production increased during storage but at a slower rate compared to pomace‐supplemented samples. Lactobacillus and Bifidobacterium species produce SCFAs via pyruvate fermentation (Pessione 2012). *Lactobacillus acidophilus* has also been shown to synthesize SCFAs in MRS medium in the presence of prebiotics (Chang et al. 2021; Farooq et al. 2013). Different prebiotics have unique properties and health benefits. These compounds resist digestion in the upper gastrointestinal tract and reach the colon intact, where they serve as fermentation substrates for beneficial gut microbes (Obayomi et al. 2024). Both in vivo and in vitro studies have demonstrated that prebiotics and monosaccharides enhance gut microbiota by improving probiotic activity and increasing SCFA production (Ban et al. 2020; Xiang et al. 2024; Zhuang et al. 2024). Moreover, dietary fiber structure—including solubility, degree of polymerization, branching, and fermentability—plays a critical role in these effects (Coste et al. 2015).

### 3.6 Sensory Evaluation of Yogurt Samples

**FIGURE 4:**
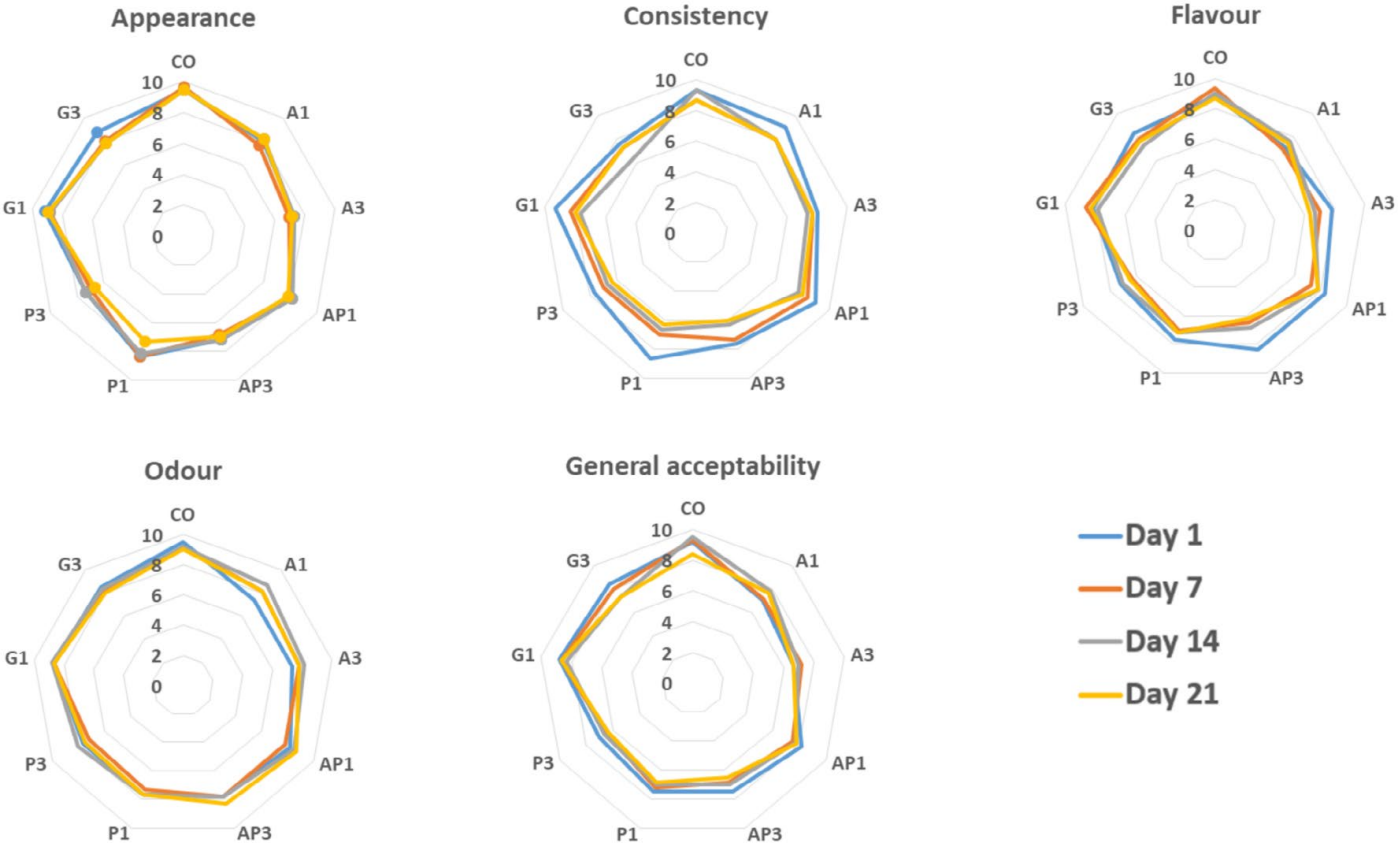
Sensory evaluation scores (scale 0–10) of yogurt samples during storage. CO: control sample without pomace addition. A1, A3: yogurt with 1% and 3% apple pomace; AP1, AP3: 1% and 3% apricot pomace; P1, P3: 1% and 3% peach pomace; G1, G3: 1% and 3% grape pomace. Sensory attributes include appearance, consistency, flavour, odour, and overall acceptability. CO: control sample without pomace addition. Data represent mean values of panelist scores evaluated over a 21‐day storage period.

Figure 4 indicates the results of the sensory evaluation of yogurt samples. The control sample consistently scored the highest in appearance throughout storage (*p* < 0.05), together with the grape pomace yogurt. Yogurts with 3% apricot, peach, and apple pomace had the lowest appearance scores, with no significant changes during storage (*p* > 0.05). On the initial day of storage, there are no noticeable variations among the samples regarding their structure and consistency scores (*p* > 0.05). During storage, samples with 3% pomace content had lower structure scores than samples with 1% pomace content; however, this difference was not statistically significant. Marand et al. (2020) reported that a significantly lower textural score at a higher concentration level (5%) could be attributed to the granularity produced by insoluble linseed powder particles and poor mouthfeel. Similarly, in our study, high pomace products produced an unpleasant mouthfeel due to their granularity. Bertolino et al. (2015) emphasized the negative impact of fiber‐rich supplementation on the structure of yogurt. In the later days of storage, yogurt with added peach pomace scored lower than the others. Apricot and grape pomace yogurts were initially more liked after the control sample. However, increasing pomace levels (especially 3% peach pomace) reduced taste scores. The 3% apricot pomace yogurt showed a considerable drop in taste evaluations during storage (*p* < 0.05), likely linked to deteriorating consistency. Jambi (2018) noted that the taste scores of yogurt containing more than 3% palm kernel powder decreased compared to the control yogurt. Bacterial proteolytic activity and increased acid generation could explain the considerable decrease in sensory qualities during storage (Bakirci and Kavaz 2008).

Grape and 1% apricot pomace yogurts scored higher in odor compared to other pomace samples, with no significant changes during storage (*p* > 0.05). Figure 4 indicates the overall acceptance scores of probiotic yogurt samples containing fruit pomace. Yogurts with 1% pomace (apple, apricot, peach, grape) were more preferred than those with 3% pomace. Higher pomace levels reduced overall acceptability, likely due to negative effects on texture and flavor. In conclusion, adding 1% of fruit pomace (grape, apple, apricot, peach) to yogurt is better received by consumers, while higher pomace levels negatively impact sensory attributes and acceptability.

## 4 Conclusion

This study demonstrates the potential of fruit juice processing by‐products—particularly apple and peach pomaces—as functional ingredients in probiotic yogurt formulations. These pomaces contributed to improved water‐holding capacity, texture, and overall sensory quality, while also enhancing probiotic viability and short‐chain fatty acid (SCFA) production. Among the samples, apple pomace showed the highest SCFA production and a rich monosaccharide composition, while grape pomace was most preferred in sensory evaluation. These results emphasize the importance of selecting appropriate fruit pomace types based on both technological and nutritional functionality. The findings support the sustainable utilization of fruit processing wastes in the dairy industry, contributing to product innovation and health promotion.

Future research should aim to deepen the understanding of dietary fiber interactions with gut microbiota and their health implications, while also confirming the findings of this study through in vivo animal models and human trials, and further exploring the complex diet–microbiome–host interactions.

## Author Contributions

**Melike Demirkol:** conceptualization (equal), data curation (equal), formal analysis (equal), investigation (equal), methodology (equal), visualization (equal), writing – original draft (equal), writing – review and editing (equal). **Zekai Tarakçi:** conceptualization (equal), data curation (equal), formal analysis (equal), investigation (equal), methodology (equal), writing – original draft (equal), writing – review and editing (equal).

## Ethics Statement

For the in vitro fecal fermentation analysis of the study, approval was obtained from Ordu University Clinical Research Ethics Committee for compliance with the ethical principles and rules of the research (code: 2019/115).

## Conflicts of Interest

The authors declare no conflicts of interest.

## Supporting information


**Table S1.** Comparison of monosaccharide composition of fibers extracted from pomace and yogurt.

## Data Availability

Data will be made available on request.

## References

[fsn370612-bib-0001] Abdel‐Hamid, M. , E. Romeih , Z. Huang , T. Enomoto , L. Huang , and L. Li . 2020. “Bioactive Properties of Probiotic Set‐Yogurt Supplemented With Siraitia Grosvenorii Fruit Extract.” Food Chemistry 303: 125400. 10.1016/j.foodchem.2019.125400. 31470275

[fsn370612-bib-0002] Archana, A.K.G. , A. Noumani , D.K. Panday , et al. 2024. “Gut Microbiota Derived Short‐Chain Fatty Acids in Physiology and Pathology: An Update.” Cell Biochemistry and Function 42, no. 7: e4108. 10.1002/cbf.4108. 39228159

[fsn370612-bib-0003] Assocıatıon Of Analytıcal Cereal Chemısts (AACC) . 2000. “AACC International Approves New Dietary Fiber Definition.” http://www.aaccnet.org/initiatives/definitions/Pages/DietaryFiber.aspx.

[fsn370612-bib-0004] Bakirci, I. , and A. Kavaz . 2008. “An Investigation of Some Properties of Banana Yogurts Made With Commercial ABT‐2 Starter Culture During Storage.” International Journal of Dairy Technology 61, no. 3: 270–276. 10.1111/j.1471-0307.2008.00409.x.

[fsn370612-bib-0005] Ban, Q. , J. Cheng , X. Sun , et al. 2020. “Effects of a Synbiotic Yogurt Using Monk Fruit Extract as Sweetener on Glucose Regulation and Gut Microbiota in Rats With Type 2 Diabetes Mellitus.” Journal of Dairy Science 103, no. 4: 2956–2968. 10.3168/jds.2019-17700. 32089310

[fsn370612-bib-0006] Bertolino, M. , S. Belviso , B. Dal Bello , et al. 2015. “Influence of the Addition of Different Hazelnut Skins on the Physicochemical, Antioxidant, Polyphenol and Sensory Properties of Yogurt.” LWT‐ Food Science and Technology 63, no. 2: 1145–1154. 10.1016/j.lwt.2015.03.113.

[fsn370612-bib-0007] Calabuig‐Jiménez, L. , L. I. Hinestroza‐Córdoba , C. Barrera , L. Seguí , and N. Betoret . 2022. “Effects of Processing and Storage Conditions on Functional Properties of Powdered Blueberry Pomace.” Sustainability 14, no. 3: 1839. 10.3390/su14031839.

[fsn370612-bib-0008] Chang, Y. H. , C. H. Jeong , W. N. Cheng , et al. 2021. “Quality Characteristics of Yogurts Fermented With Short‐Chain Fatty Acid‐Producing Probiotics and Their Effects on Mucin Production and Probiotic Adhesion Onto Human Colon Epithelial Cells.” Journal of Dairy Science 104, no. 7: 7415–7425. 10.3168/jds.2020-19820. 33814147

[fsn370612-bib-0009] Chen, G. , Z. Zeng , M. Xie , et al. 2022. “Fermentation Characteristics and Probiotic Activity of a Purified Fraction of Polysaccharides From Fuzhuan Brick Tea.” Food Science and Human Wellness 11, no. 3: 727–737. 10.1016/j.fshw.2021.12.030.

[fsn370612-bib-0010] Chouchouli, V. , N. Kalogeropoulos , S. J. Konteles , E. Karvela , D. P. Makris , and V. T. Karathanos . 2013. “Fortification of Yoghurts With Grape ( *Vitis vinifera* ) Seed Extracts.” LWT‐ Food Science and Technology 53, no. 2: 522–529. 10.1016/j.lwt.2013.03.008.

[fsn370612-bib-0011] Coste, O. , E.‐j. Malta , J. C. López , and C. Fernández‐Díaz . 2015. “Production of Sulfated Oligosaccharides From the Seaweed Ulva sp. Using a New Ulvan‐Degrading Enzymatic Bacterial Crude Extract.” Algal Research 10: 224–231. 10.1016/j.algal.2015.05.014.

[fsn370612-bib-0012] Cui, J. , Y. Li , Q. Wang , et al. 2017. “Production, Purification and Analysis of the Isomalto‐Oligosaccharides From Chinese Chestnut ( *Castanea mollissima* Blume) and the Prebiotics Effects of Them on Proliferation of Lactobacillus.” Food and Bioproducts Processing 106: 75–81. 10.1016/j.fbp.2017.08.003.

[fsn370612-bib-0013] Cui, J. , Y. Lian , C. Zhao , et al. 2019. “Dietary Fibers From Fruits and Vegetables and Their Health Benefits via Modulation of Gut Microbiota.” Comprehensive Reviews in Food Science and Food Safety 18, no. 5: 1514–1532. 33336908 10.1111/1541-4337.12489

[fsn370612-bib-0014] Davis, L. , I. Martinez , J. Walter , and R. Hutkins . 2010. “A Dose Dependent Impact of Prebiotic Galactooligosaccharides on the Intestinal Microbiota of Healthy Adults.” International Journal of Food Microbiology 144, no. 2: 285–292. 10.1016/j.ijfoodmicro.2010.10.007. 21059476

[fsn370612-bib-0015] de Moraes Crizel, T. , A. Jablonski , A. de Oliveira Rios , R. Rech , and S. H. Flôres . 2013. “Dietary Fiber From Orange Byproducts as a Potential Fat Replacer.” LWT‐ Food Science and Technology 53, no. 1: 9–14. 10.1016/j.lwt.2013.02.002.

[fsn370612-bib-0016] Demirkol, M. 2021. “Meyve Suyu İşleme Atıklarından Diyet Liflerinin İzolasyonu ve Yoğurt Üretiminde Kullanımı.”

[fsn370612-bib-0017] Demirkol, M. , and Z. Tarakci . 2018. “Effect of Grape ( *Vitis labrusca* L.) Pomace Dried by Different Methods on Physicochemical, Microbiological and Bioactive Properties of Yoghurt.” LWT 97: 770–777. 10.1016/j.lwt.2018.07.058.

[fsn370612-bib-0018] Farooq, U. , M. Mohsin , X. Liu , and H. Zhang . 2013. “Enhancement of Short Chain Fatty Acid Production From Millet Fibres by Pure Cultures of Probiotic Fermentation.” Tropical Journal of Pharmaceutical Research 12, no. 2: 189–194. 10.4314/tjpr.v12i2.9.

[fsn370612-bib-0019] Fernandez, M. A. , and A. Marette . 2017. “Potential Health Benefits of Combining Yogurt and Fruits Based on Their Probiotic and Prebiotic Properties.” Advances in Nutrition 8, no. 1: 155S–164S. 10.3945/an.115.011114. 28096139 PMC5227968

[fsn370612-bib-0020] Figuerola, F. , M. L. Hurtado , A. M. Estévez , I. Chiffelle , and F. Asenjo . 2005. “Fibre Concentrates From Apple Pomace and Citrus Peel as Potential Fibre Sources for Food Enrichment.” Food Chemistry 91, no. 3: 395–401. 10.1016/j.foodchem.2004.04.036.

[fsn370612-bib-0021] Ge, Q. , H.‐q. Li , Z.‐y. Zheng , et al. 2022. “In Vitro Fecal Fermentation Characteristics of Bamboo Insoluble Dietary Fiber and Its Impacts on Human Gut Microbiota.” Food Research International 156: 111173. 10.1016/j.foodres.2022.111173. 35651096

[fsn370612-bib-0022] Geraldi, M. V. , F. L. Tulini , V. M. Souza , and E. C. De Martinis . 2018. “Development of Yoghurt With Juçara Pulp (Euterpe Edulis M.) and the Probiotic *Lactobacillus acidophilus* La5.” Probiotics and Antimicrobial Proteins 10: 71–76. 10.1007/s12602-017-9280-z. 28432677

[fsn370612-bib-0023] Göçer, E. M. Ç. , F. Ergin , A. A. Arslan , and A. Küçükçetin . 2016. “Farklı İnkübasyon Sıcaklığı ile İnkübasyon Sonlandırma pH'sının Probiyotik Yoğurdun Fizikokimyasal ve Mikrobiyolojik Özellikleri Üzerine Etkisi.” Akademik Gıda 14, no. 4: 341–350. 10.24323/akademik-gida.370105.

[fsn370612-bib-0024] Gouw, V. P. , J. Jung , and Y. Zhao . 2017. “Functional Properties, Bioactive Compounds, and In Vitro Gastrointestinal Digestion Study of Dried Fruit Pomace Powders as Functional Food Ingredients.” LWT 80: 136–144. 10.1016/j.lwt.2017.02.015.

[fsn370612-bib-0025] Güler‐Akın, M. B. , B. Goncu , and M. S. Akın . 2018. “Some Properties of Bio‐Yogurt Enriched With Cellulose Fiber.” Advances in Microbiology 8, no. 1: 54–64. 10.4236/aim.2018.81005.

[fsn370612-bib-0026] Hamaker, B. R. , and Y. E. Tuncil . 2014. “A Perspective on the Complexity of Dietary Fiber Structures and Their Potential Effect on the Gut Microbiota.” Journal of Molecular Biology 426, no. 23: 3838–3850. 10.1016/j.jmb.2014.07.028. 25088686

[fsn370612-bib-0027] Hassan, F. A. , A. Ismail , A. A. Hamid , A. Azlan , and S. H. Al‐sheraji . 2011. “Characterisation of Fibre‐Rich Powder and Antioxidant Capacity of Mangifera Pajang K. Fruit Peels.” Food Chemistry 126, no. 1: 283–288. 10.1016/j.foodchem.2010.11.019.

[fsn370612-bib-0028] Helal, A. , N. Rashid , M. Dyab , M. Otaibi , and T. Alnemr . 2018. “Enhanced Functional, Sensory, Microbial and Texture Properties of Low‐Fat Set Yogurt Supplemented With High‐Density Inulin.” Journal of Food Processing & Beverages 6, no. 1: 1–11. 10.13188/2332-4104.1000020.

[fsn370612-bib-0029] Helland, M. H. , T. Wicklund , and J. A. Narvhus . 2004. “Growth and Metabolism of Selected Strains of Probiotic Bacteria in Milk‐ and Water‐Based Cereal Puddings.” International Dairy Journal 14, no. 11: 957–965. 10.1016/j.idairyj.2004.03.008.

[fsn370612-bib-0030] International, A . 2000. Official Methods of Analysis of AOAC International. Vol. 17. AOAC international.

[fsn370612-bib-0031] Isleten, M. , and Y. Karagul‐Yuceer . 2006. “Effects of Dried Dairy Ingredients on Physical and Sensory Properties of Nonfat Yogurt.” Journal of Dairy Science 89, no. 8: 2865–2872. 10.3168/jds.s0022-0302(06)72559-0. 16840602

[fsn370612-bib-0032] Jambi, H. A. 2018. “Evaluation of Physio‐Chemical and Sensory Properties of Yogurt Prepared With Date Pits Powder.” Current Science International 7, no. 1: 1–9.

[fsn370612-bib-0033] Jia, R. , H. Chen , H. Chen , and W. Ding . 2016. “Effects of Fermentation With *Lactobacillus rhamnosus* GG on Product Quality and Fatty Acids of Goat Milk Yogurt.” Journal of Dairy Science 99, no. 1: 221–227. 10.3168/jds.2015-10114. 26601583

[fsn370612-bib-0034] Koropatkin, N. M. , E. A. Cameron , and E. C. Martens . 2012. “How Glycan Metabolism Shapes the Human Gut Microbiota.” Nature Reviews Microbiology 10, no. 5: 323–335. 10.1038/nrmicro2746. 22491358 PMC4005082

[fsn370612-bib-0035] Lario, Y. , E. Sendra , J. Garcıa‐Pérez , et al. 2004. “Preparation of High Dietary Fiber Powder From Lemon Juice By‐Products.” Innovative Food Science & Emerging Technologies 5, no. 1: 113–117. 10.1016/j.ifset.2003.08.001.

[fsn370612-bib-0036] Larrauri, J. 1999. “New Approaches in the Preparation of High Dietary Fibre Powders From Fruit By‐Products.” Trends in Food Science & Technology 10, no. 1: 3–8. 10.1016/s0924-2244(99)00016-3.

[fsn370612-bib-0037] Laufenberg, G. , B. Kunz , and M. Nystroem . 2003. “Transformation of Vegetable Waste Into Value Added Products::(A) the Upgrading Concept;(B) Practical Implementations.” Bioresource Technology 87, no. 2: 167–198. 10.1016/s0960-8524(02)00167-0. 12765356

[fsn370612-bib-0038] Lee, W.‐J. , and J. Lucey . 2010. “Formation and Physical Properties of Yogurt.” Asian‐Australasian Journal of Animal Sciences 23, no. 9: 1127–1136. 10.5713/ajas.2010.r.05.

[fsn370612-bib-0039] Lei, W. , Q. Chen , Y. Liu , et al. 2023. “Partial Purification, Characterization, and Application of Exopolysaccharides Produced by *Lactobacillus plantarum* NS1905E in Yogurt.” Journal of Food Biochemistry 2023: 8828565. 10.1155/2023/8828565.

[fsn370612-bib-0040] Li, X. R. , C. J. Liu , X. D. Tang , et al. 2020. “Gut Microbiota Alterations From Three‐Strain Yogurt Formulation Treatments in Slow‐Transit Constipation.” Canadian Journal of Infectious Diseases and Medical Microbiology 2020: 4583973. 10.1155/2020/4583973. 32148595 PMC7049856

[fsn370612-bib-0041] Lv, J.‐S. , X.‐Y. Liu , X.‐P. Zhang , and L.‐S. Wang . 2017. “Chemical Composition and Functional Characteristics of Dietary Fiber‐Rich Powder Obtained From Core of Maize Straw.” Food Chemistry 227: 383–389. 10.1016/j.foodchem.2017.01.078. 28274447

[fsn370612-bib-0042] Mani‐López, E. , E. Palou , and A. López‐Malo . 2014. “Probiotic Viability and Storage Stability of Yogurts and Fermented Milks Prepared With Several Mixtures of Lactic Acid Bacteria.” Journal of Dairy Science 97, no. 5: 2578–2590. 10.3168/jds.2013-7551. 24745665

[fsn370612-bib-0043] Marand, M. A. , S. Amjadi , M. A. Marand , L. Roufegarinejad , and S. M. Jafari . 2020. “Fortification of Yogurt With Flaxseed Powder and Evaluation of Its Fatty Acid Profile, Physicochemical, Antioxidant, and Sensory Properties.” Powder Technology 359: 76–84. 10.1016/j.powtec.2019.09.082.

[fsn370612-bib-0044] Martinez, R. , P. Torres , M. A. Meneses , J. G. Figueroa , J. A. Perez‐Alvarez , and M. Viuda‐Martos . 2012. “Chemical, Technological and In Vitro Antioxidant Properties of Mango, Guava, Pineapple and Passion Fruit Dietary Fibre Concentrate.” Food Chemistry 135, no. 3: 1520–1526. 10.1016/j.foodchem.2012.05.057. 22953888

[fsn370612-bib-0045] Mazhar, M. , Y. Zhu , and L. Qin . 2023. “The Interplay of Dietary Fibers and Intestinal Microbiota Affects Type 2 Diabetes by Generating Short‐Chain Fatty Acids.” Food 12, no. 5: 1023. 10.3390/foods12051023. PMC10001013 36900540

[fsn370612-bib-0046] Mortazavian, A. , M. Ehsani , S. Mousavi , et al. 2006. “Preliminary Investigation of the Combined Effect of Heat Treatment and Incubation Temperature on the Viability of the Probiotic Micro‐Organisms in Freshly Made Yogurt.” International Journal of Dairy Technology 59, no. 1: 8–11. 10.1111/j.1471-0307.2006.00216.x.

[fsn370612-bib-0047] Ndeh, D. , A. Rogowski , A. Cartmell , et al. 2017. “Complex Pectin Metabolism by Gut Bacteria Reveals Novel Catalytic Functions.” Nature 544, no. 7648: 65–70. 28329766 10.1038/nature21725 PMC5388186

[fsn370612-bib-0048] Obayomi, O. V. , A. F. Olaniran , and S. O. Owa . 2024. “Unveiling the Role of Functional Foods With Emphasis on Prebiotics and Probiotics in Human Health: A Review.” Journal of Functional Foods 119: 106337. 10.1016/j.jff.2024.106337.

[fsn370612-bib-0049] Oh, N. S. , J. Y. Lee , J. Y. Joung , et al. 2016. “Microbiological Characterization and Functionality of Set‐Type Yogurt Fermented With Potential Prebiotic Substrates Cudrania Tricuspidata and *Morus alba* L. Leaf Extracts.” Journal of Dairy Science 99, no. 8: 6014–6025. 10.3168/jds.2015-10814. 27236762

[fsn370612-bib-0050] Paseephol, T. , D. M. Small , and F. Sherkat . 2008. “Rheology and Texture of Set Yogurt as Affected by Inulin Addition.” Journal of Texture Studies 39, no. 6: 617–634. 10.1111/j.1745-4603.2008.00161.x.

[fsn370612-bib-0051] Pessione, E. 2012. “Lactic Acid Bacteria Contribution to Gut Microbiota Complexity: Lights and Shadows.” Frontiers in Cellular and Infection Microbiology 2: 86. 10.3389/fcimb.2012.00086. 22919677 PMC3417654

[fsn370612-bib-0052] Ranadheera, C. S. , C. Evans , M. Adams , and S. Baines . 2012. “Probiotic Viability and Physico‐Chemical and Sensory Properties of Plain and Stirred Fruit Yogurts Made From Goat's Milk.” Food Chemistry 135, no. 3: 1411–1418. 10.1016/j.foodchem.2012.06.025. 22953874

[fsn370612-bib-0053] Sah, B. N. P. , T. Vasiljevic , S. McKechnie , and O. Donkor . 2016. “Physicochemical, Textural and Rheological Properties of Probiotic Yogurt Fortified With Fibre‐Rich Pineapple Peel Powder During Refrigerated Storage.” LWT‐ Food Science and Technology 65: 978–986. 10.1016/j.lwt.2015.09.027.

[fsn370612-bib-0054] Sah, B. N. P. , T. Vasiljevic , S. McKechnie , and O. N. Donkor . 2015. “Effect of Refrigerated Storage on Probiotic Viability and the Production and Stability of Antimutagenic and Antioxidant Peptides in Yogurt Supplemented With Pineapple Peel.” Journal of Dairy Science 98, no. 9: 5905–5916. 10.3168/jds.2015-9450. 26142843

[fsn370612-bib-0055] Sahni, P. , and D. Shere . 2018. “Utilization of Fruit and Vegetable Pomace as Functional Ingredient in Bakery Products: A Review.” Asian Journal of Dairy and Food Research 37, no. 3: 202–211.

[fsn370612-bib-0056] Santo, A. E. , N. Cartolano , T. Silva , et al. 2012. “Fibers From Fruit By‐Products Enhance Probiotic Viability and Fatty Acid Profile and Increase CLA Content in Yoghurts.” International Journal of Food Microbiology 154, no. 3: 135–144. 10.1016/j.ijfoodmicro.2011.12.025. 22264421

[fsn370612-bib-0057] Sayar, S. , J.‐L. Jannink , and P. J. White . 2007. “Digestion Residues of Typical and High‐β‐Glucan Oat Flours Provide Substrates for In Vitro Fermentation.” Journal of Agricultural and Food Chemistry 55, no. 13: 5306–5311. 10.1021/jf070240z. 17550267

[fsn370612-bib-0058] Sendra, E. , V. Kuri , J. Fernández‐López , E. Sayas‐Barbera , C. Navarro , and J. Pérez‐Alvarez . 2010. “Viscoelastic Properties of Orange Fiber Enriched Yogurt as a Function of Fiber Dose, Size and Thermal Treatment.” LWT‐Food Science and Technology 43, no. 4: 708–714. 10.1016/j.lwt.2009.12.005.

[fsn370612-bib-0059] Shori, A. B. 2013. “Antioxidant Activity and Viability of Lactic Acid Bacteria in Soybean‐Yogurt Made From Cow and Camel Milk.” Journal of Taibah University for Science 7, no. 4: 202–208. 10.1016/j.jtusci.2013.06.003.

[fsn370612-bib-0060] Srisuvor, N. , N. Chinprahast , C. Prakitchaiwattana , and S. Subhimaros . 2013. “Effects of Inulin and Polydextrose on Physicochemical and Sensory Properties of Low‐Fat Set Yoghurt With Probiotic‐Cultured Banana Purée.” LWT‐Food Science and Technology 51, no. 1: 30–36. 10.1016/j.lwt.2012.10.018.

[fsn370612-bib-0061] Tang, J. , X. Peng , D. M. Liu , Y. Q. Xu , J. Xiong , and J. J. Wu . 2023. “Assessment of the Safety and Probiotic Properties of *Lactobacillus delbrueckii* DMLD‐H1 Based on Comprehensive Genomic and Phenotypic Analysis.” LWT ‐ Food Science and Technology 184: 115070. 10.1016/j.lwt.2023.115070.

[fsn370612-bib-0062] Tarakçı, Z. , and E. Küçüköner . 2003. “Physical, Chemical, Microbiological and Sensory Characteristics of Some Fruit‐Flavored Yoghurt.” Yüzüncü Yıl Üniversitesi Veteriner Fakültesi Dergisi 14, no. 2: 10–14.

[fsn370612-bib-0063] Tseng, A. , and Y. Zhao . 2013. “Wine Grape Pomace as Antioxidant Dietary Fibre for Enhancing Nutritional Value and Improving Storability of Yogurt and Salad Dressing.” Food Chemistry 138, no. 1: 356–365. 10.1016/j.foodchem.2012.09.148. 23265499

[fsn370612-bib-0064] Tuncil, Y. , T. Jondiko , M. Tilley , D. Hays , and J. Awika . 2016. “Combination of Null Alleles With 7+ 9 Allelic Pair at Glu‐B1 Locus on the Long Arm of Group 1 Chromosome Improves Wheat Dough Functionality for Tortillas.” LWT‐ Food Science and Technology 65: 683–688. 10.1016/j.lwt.2015.08.074.

[fsn370612-bib-0065] Tuncil, Y. E. , C. H. Nakatsu , A. E. Kazem , et al. 2017. “Delayed Utilization of Some Fast‐Fermenting Soluble Dietary Fibers by Human Gut Microbiota When Presented in a Mixture.” Journal of Functional Foods 32: 347–357. 10.1016/j.jff.2017.03.001.

[fsn370612-bib-0066] Tuncil, Y. E. , R. D. Thakkar , S. Arioglu‐Tuncil , B. R. Hamaker , and S. R. Lindemann . 2018. “Fecal Microbiota Responses to Bran Particles Are Specific to Cereal Type and In Vitro Digestion Methods That Mimic Upper Gastrointestinal Tract Passage.” Journal of Agricultural and Food Chemistry 66, no. 47: 12580–12593. 10.1021/acs.jafc.8b03469. 30406656

[fsn370612-bib-0067] Wang, L. , H. Xu , F. Yuan , Q. Pan , R. Fan , and Y. Gao . 2015. “Physicochemical Characterization of Five Types of Citrus Dietary Fibers.” Biocatalysis and Agricultural Biotechnology 4, no. 2: 250–258. 10.1016/j.bcab.2015.02.003.

[fsn370612-bib-0068] Xiang, S. , Y. Ge , Y. Zhang , et al. 2024. “L‐Arabinose Exerts Probiotic Functions by Improving Gut Microbiota and Metabolism In Vivo and In Vitro.” Journal of Functional Foods 113: 106047. 10.1016/j.jff.2024.106047.

[fsn370612-bib-0069] Xiong, J. , J.‐j. Yu , D.‐m. Liu , et al. 2023. “A Polysaccharide From Yogurt Fermented by Lactobacillus Bulgaricus and *Streptococcus thermophilus*: Structural Characteristics and Its Alleviative Effect on DSS‐Induced Colitis in Mice.” Journal of Functional Foods 109: 105778. 10.1016/j.jff.2023.105778.

[fsn370612-bib-0070] Xue, R. , J. Liu , M. Zhang , et al. 2024. “Physicochemical, Microbiological and Metabolomics Changes in Yogurt Supplemented With Lactosucrose.” Food Research International 178: 114000. 10.1016/j.foodres.2024.114000. 38309926

[fsn370612-bib-0071] Zeng, H. , P. Chen , C. Chen , et al. 2018. “Structural Properties and Prebiotic Activities of Fractionated Lotus Seed Resistant Starches.” Food Chemistry 251: 33–40. 10.1016/j.foodchem.2018.01.057. 29426421

[fsn370612-bib-0072] Zhang, C. , T. Fang , L. Shi , et al. 2024. “The Synbiotic Combination of Probiotics and Inulin Improves NAFLD Though Modulating Gut Microbiota.” Journal of Nutritional Biochemistry 125: 109546. 10.1016/j.jnutbio.2023.109546. 38072206

[fsn370612-bib-0073] Zhuang, M. , G. Li , S. Ke , A. Wang , X. Wang , and Z. Zhou . 2024. “A Higher Butyrate‐Generation Capacity of Water‐Extractable Substrates From Wheat Bran as Evidenced by In Vitro and In Vivo Fermentation.” Food Bioscience 59: 103867. 10.1016/j.fbio.2024.103867.

