# Supplementary material for: Utilization of Fruit Juice Processing Wastes as Prebiotic Ingredients in Probiotic Yogurt: Effects on Microbial Short Chain Fatty Acid Production
Source: Food Sci Nutr. 2025 Jul 14;13(7):e70612. doi: 10.1002/fsn3.70612 (PMC12257124; doi:10.1002/fsn3.70612)
Supplement: Supplementary file 1 — Table S1. Comparison of monosaccharide composition of fibers extracted from pomace and yogurt. [file FSN3-13-e70612-s001.docx]

Table S1. Comparison of monosaccharide composition of fibers extracted from pomace and yogurt

| Dietary fiber |  | Rhamnose | Arabinose | Xylose | Mannose | Galaktose | Glucose | Uronic acid |
| --- | --- | --- | --- | --- | --- | --- | --- | --- |
| YOGURT DF | Apple | 0.11 ± 0.05^B^ | 1.41 ± 0.47^B^ | 0.52 ± 0.12^B^ | 0.32 ± 0.09^AB^ | 3.39 ± 1.22^A^ | 8.05 ± 2.35^AB^ | 4.10 ± 0.42^C^ |
| 3% | Apricot | 0.02 ± 0.02^B^ | 0.25 ± 0.09^C^ | 0.16 ± 0.08^B^ | 0.05 ± 0.01^B^ | 1.74 ± 0.45^AB^ | 2.19 ± 0.62^D^ | 3.75 ± 0.02^CD^ |
|  | Peach | 0.04 ± 0.07^B^ | 0.38 ± 0.35^C^ | 0.17 ± 0.14^B^ | 0.08 ± 0.07^B^ | 1.58 ± 1.08^AB^ | 2.49 ± 1.76^D^ | 3.07 ± 0.22^D^ |
|  | Grape | - | 0.10 ± 0.04^C^ | 0.37 ± 0.39^B^ | 0.08 ± 0.06^B^ | 0.76 ± 0.12^B^ | 1.75 ± 0.32^D^ | 2.09 ± 0.07^E^ |
|  | Control | 0.02 ± 0.01^B^ | - | - | 0.01 ± 0.01^B^ | 2.35 ± 0.28^AB^ | 2.16 ± 0.28^D^ | 0.30 ± 0.05^F^ |
| TDF | Apple | 0.03 ± 0.01^B^ | 0.40 ± 0.03^C^ | 0.17 ± 0.02^B^ | 0.08 ± 0.01^B^ | 0.26 ± 0.00^B^ | 2.03 ± 0.12^D^ | 10.34 ± 0.70^A^ |
|  | Apricot | - | 0.16 ± 0.08^C^ | 0.11 ± 0.05^B^ | 0.02 ± 0.02^B^ | 0.08 ± 0.05^B^ | 0.75 ± 0.33^DE^ | 8.15 ± 0.28^AB^ |
|  | Peach | 0.27 ± 0.00^A^ | 2.81 ± 0.33^A^ | 1.40 ± 0.18^A^ | 0.47 ± 0.02^A^ | 1.48 ± 0.09^AB^ | 8.35 ± 0.22^A^ | 8.27 ± 1.32^AB^ |
|  | Grape | 0.05 ± 0.04^B^ | 0.27 ± 0.15^C^ | 0.36 ± 0.00^B^ | 0.28 ± 0.22^AB^ | 0.32 ± 0.20^B^ | 4.98 ± 3.05^C^ | 5.68 ± 0.40^B^ |

^A-E^ Values with different letters in the same column differ from one another at p<0.05 level. YOGURT DF; yoğurt dietary fiber, TDF; pomace dietary fiber, Control; plain yogurt.
